# Intra-abdominal hypertension in cardiac surgery patients: a multicenter observational sub-study of the Accuryn registry

**DOI:** 10.1007/s10877-022-00878-2

**Published:** 2022-06-13

**Authors:** Ashish K. Khanna, Steven Minear, Andrea Kurz, Vanessa Moll, Kelly Stanton, Leina Essakalli, Amit Prabhakar, Predict AKI Group, Lynnette C. Harris, Nia Sweatt, Kelsey Flores, Brandon Reeves, Bruce Cusson, Lillian Nosow, Jessica Fanelli, Lauren Sands, Jacob Fowler, Easton Howard, Samuel Robinson, Anthony Wachnik, Madeline Fram, Rohesh Fernando, Chandrika Garner, Bryan Marchant, Benjamin Morris, Amit Saha, Katherine Egan, Bev Ann Blackwell

**Affiliations:** 1 Department of Anesthesiology, Section on Critical Care Medicine, Wake Forest School of Medicine, Atrium Health Wake Forest Baptist Medical Center, Winston-Salem, NC USA; 2 Outcomes Research Consortium, Cleveland, OH USA; 3 Department of Anesthesiology, Cleveland Clinic Florida, Weston Hospital, 2950 Cleveland Clinic Boulevard, Weston, FL USA; 4 Departments of General Anesthesiology and Outcomes Research, Cleveland Clinic, Anesthesiology Institute, 9500 Euclid Avenue/E-31, Cleveland, OH USA; 5 Department of Anesthesiology, Emergency Medicine and Intensive Care Medicine, Medical University Graz, Graz, Austria; 6 Potrero Medical, Hayward, CA USA; 7 Department of Anesthesiology, Division of Critical Care Medicine, Emory University School of Medicine, Atlanta, GA USA

**Keywords:** Intra-abdominal pressure; IAP, Intra-abdominal hypertension; IAH, Cardiac surgery, Perioperative, Abdominal compartment syndrome, Real-time monitoring

## Abstract

Intra-abdominal hypertension (IAH) is frequently present in the critically ill and is associated with increased morbidity and mortality. Conventionally, intermittent ‘spot-check’ manual measurements of bladder pressure in those perceived as high risk are used as surrogates for intra-abdominal pressure (IAP). True patterns of IAH remain unknown. We explored the incidence of IAH in cardiac surgery patients and describe the intra-and postoperative course of IAP using a novel, high frequency, automated bladder pressure measurement system. Sub-analysis of a prospective, multicenter, observational study (NCT04669548) conducted in three large academic medical centers. Continuous urinary output (CUO) and IAP measurements were observed using the Accuryn Monitoring System (Potrero Medical, Hayward, CA). Data collected included demographics, hemodynamic support, and high-frequency IAP and CUO. One Hundred Thirty-Seven cardiac surgery patients were analyzed intraoperatively and followed 48 h postoperatively in the intensive care unit. Median age was 66.4 [58.3, 72.0] years, and 61% were men. Median Foley catheter dwell time was 56.0 [46.8, 77.5] hours, and median baseline IAP was 6.3 [4.0, 8.1] mmHg. 93% (128/137) of patients were in IAH grade I, 82% (113/137) in grade II, 39% (53/137) in grade III, and 5% (7/137) in grade IV for at least 12 cumulative hours. For maximum consecutive duration of IAH, 84% (115/137) of patients spent at least 12 h in grade I, 62% (85/137) in grade II, 18% (25/137) in grade III, and 2% (3/137) in grade IV IAH. During the first 48 h after cardiac surgery, IAH is common and persistent. Improved and automated monitoring of IAP will increase the detection of IAH—which normally would remain undetected using traditional intermittent monitoring methods.

**Supplementary Information:**

The online version contains supplementary material available at 10.1007/s10877-022-00878-2.

## Introduction

Intra-abdominal hypertension (IAH) is frequent in the critically ill [1–4]. On admission IAH is present in 25–30% and an IAP above 12 mmHg in 50% of patients within the first week of ICU stay [5]. IAH is associated with increased morbidity and mortality [1, 3, 6]. IAH is defined as sustained intra-abdominal pressures (IAP) ≥ 12 mmHg. A sustained IAP > 20 mmHg that is associated with new-onset organ dysfunction or failure defines abdominal compartment syndrome (ACS) [7]. Systemic perioperative hypotension defined as mean arterial pressure (MAP) decreases abdominal perfusion pressure (APP) based on the relationship where APP = MAP − IAP and is strongly associated with an increased risk of organ system injury [8–10]. APP is further compromised by IAH and increases the risk for AKI [11–14]. As a consequence of its effect on abdominal organs, patients with IAH display 28- and 90-day mortalities that are twice as high compared to patients without IAH (28-day- 27.1% vs. 10.8% and 90-day-mortality 36.7% vs. 16.3%) [1].

Risk factors for IAH and ACS include massive transfusion or fluid resuscitation, hypothermia, acidosis, and hypotension [7]. In general terms, these risk factors can be separated into those affecting abdominal compliance, increased intraluminal contents, increased intra-abdominal contents and capillary leak leading to extravascular and fluid accumulation [15, 16]. Current practice patterns in the ICU systems the world over dictate manual, intermittent IAP monitoring only in patients with high clinical suspicion and two or more risk factors for IAH. Various techniques have been described in the literature that permit the trans-bladder monitoring technique recommended by the Abdominal Compartment Society (WSACS) [17], but all require the instillation of normal saline into the bladder. These methods use the hydrostatic column technique and thus are cumbersome, labor-intensive, prone to inter-user variability, and leave large gaps of unmonitored time. While continuous IAP measurement has previously been described either by 3-way Foley catheter or via measuring gastric or direct intraperitoneal pressure [18, 19], none of these techniques have been clinically adopted. The ability to frequently and automatically measure bladder pressure through a Foley catheter was, in the past, not available. The Accuryn Monitoring System (Potrero Medical, Hayward, CA), which the FDA cleared for monitoring and measuring IAP, continuous urinary output (CUO), and core body temperature, has been recently introduced [20, 21]. This novel device measures bladder pressure through a semi-flaccid balloon containing a pressure sensor at the tip of a Foley catheter at 100 Hz. The system thereby enables high-fidelity, high-resolution monitoring, and it can be utilized to detect IAH severity and duration across a range of patient scenarios.

Cardiac surgery patients have multiple risk factors for the development of IAH during surgery and afterward in the ICU. However, IAH has not been well described in this patient population, with only a few small single-center studies reporting an incidence of IAH of 33–46% [22–29]. We present pilot data from an ongoing prospective, multicenter observational study (NCT04669548). Our primary objective was to describe the incidence, duration and severity of IAH above a priori determined pre-established thresholds, i.e., IAH grade I–IV from anesthesia start to the first 48 postoperative hours in the ICU. Furthermore, we also describe the longitudinal course of IAP and associated UO in these patients.

## Materials and methods

### Study design

This was an observational, multicenter pilot study approved at all participating sites (IRB00099580, IRB00069008, FLA 20-044) and registered with ClinicalTrials.gov (NCT04669548). The primary objective of the large observational multicenter study (NCT04669548) is to track and analyze data streams [IAP, urine output, temperature] captured using the Accuryn® Monitoring System and correlate these values to the occurrence of AKI [KDIGO, AKI defined as AKI stage > 0] following cardiovascular surgical intervention(s). The secondary objective is to determine the occurrence of intraoperative and/or postoperative intra-abdominal hypertension (IAH), analyze contributing factors to the development of IAH, and analyze associations of IAH with organ dysfunctions such as acute kidney injury and other clinical outcomes (e.g., ICU length of stay, required hemodialysis, readmission).

Informed written consent was acquired and verified for all patients at Wake Forest (retrospective written consent) and at Cleveland Clinic Florida (prospective written consent). Emory University School of Medicine’s IRB granted a waiver of consent.

Data included in the current analysis were collected between March 25th and October 1st, 2019 (Emory) and December 16th, 2020, and April 15th, 2021 (Wake Forest and Cleveland Clinic Florida) and represent pilot data from an ongoing large registry currently enrolling at centers across the country. We prospectively enrolled elective and emergent adult cardiac surgery patients at three large academic medical centers in the USA, undergoing a variety of on and off-pump cardiac surgical procedures needing open sternotomy. The Accuryn Monitoring System intra-abdominal pressure and CUO Foley catheter was inserted after induction of anesthesia and stayed in place along with the bedside measurement system util the patient stayed in the intensive care unit (ICU) or did not need a urinary catheter (whichever came first). While CUO data displayed as continuous output per unit time was used as part of routine care, IAP was not and was recorded passively and extracted retrospectively from the system.

### Data collection

Data collected included high-resolution urine output, intra-abdominal pressure, and core body temperature. The Accuryn Monitoring System measures bladder pressure at 100 Hz through a semi-flaccid balloon containing a pressure sensor at the tip of a Foley catheter. Intra-abdominal pressure was measured within the bladder at 100 Hz and re-sampled to 1 Hz by taking the first measurement every second. A 30 s running minimum was applied to calculated end-expiratory pressure, and a 10 min running median was then applied to stabilize the calculation to outliers (such as coughing, straining). The Accuryn Monitoring System clears urine from the tubing lines automatically on a regular basis (active drain line clearance). This ensures that all urine is collected and not remaining in the bladder or the drain lines. Urine output was sampled approximately 10 s after each active drain line clearance event to ensure that all urine was accounted for, and the rate was then linearly interpolated between clearance events. IAP and CUO data were then re-sampled at 15 min intervals. Baseline IAP was defined as the average bladder pressure collected over the first hour of Accuryn Monitoring System data for each patient. The Accuryn Foley catheters were inserted after induction of anesthesia and remained in place at the clinical team’s discretion.

**Fig. 1:**
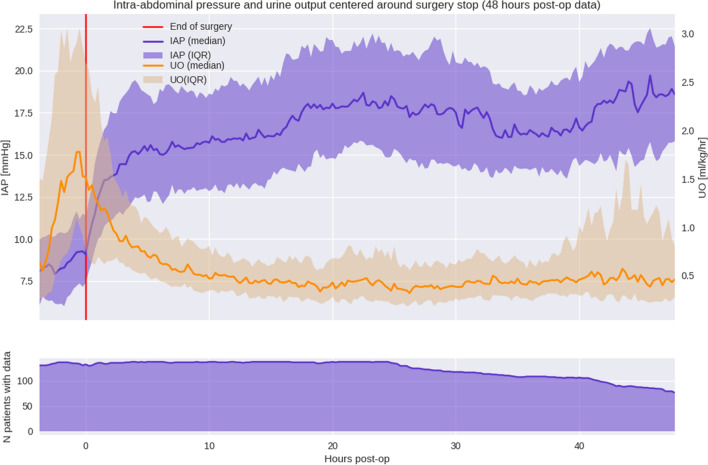
Intra-abdominal pressure and urinary output intra-operatively and for the first 48 postoperative hours. The red line represents surgery end. *IAP* intra-abdominal pressure; *UO* urinary output; *IQR* interquartile range; *mmHg* millimeter Hydrargyrum (Mercury); *ml/kg/h* milliliter/kilogram/hour

We collected additional data such as age, gender, race and ethnicity, body mass index (BMI), vasopressors, inotropes, transfusions and extubation times from the electronic medical record. Data were deidentified on-site. BMI was further used to classify overweight and obesity according to the Center of Disease Control (CDC) [30]. Accuryn Monitoring System data (IAP, CUO) were related to EMR data using a deidentified patient identifier. We excluded patients if they had less than 24 h of data from the Accuryn Monitoring System or if data variables were missing (extubation times were exempt from this missing data exclusion). Time to extubation was calculated from surgery end to better align with Fig. 1. We performed a separate analysis to describe IAP in the patients in whom the Accuryn Monitoring System was in place for less than 24 h. Data collection from the Accuryn Monitoring System was seized when Foley catheters were pulled, or patients left the ICU.

### Outcome measures

We evaluated the incidence and severity of IAH above the established thresholds, i.e., IAH grade I–IV from anesthesia start until 48 h postoperatively. Furthermore, we assessed the duration of IAH at different severity grades.

### Statistical analysis

Continuous variables are presented as medians with interquartile ranges following in brackets, [IQR], and categorical variables as percentages followed by the ratio in parentheses (count/total).

IAH was defined as previously described by the WSACS [7]. However, WSACS currently does not grade IAP in ranges 15–16 mmHg and 20–21 mmHg. The Accuryn Monitoring System delivers decimal precision IAP; thus, for the current study, we used the following convention to grade the severity of IAH: grades I–IV were defined as IAP ≥ 12 and < 15, ≥ 15 and < 20, and ≥ 20 and < 25 and ≥ 25 mmHg, respectively.

Times were sampled to the nearest 15 min allowing alignment and comparison of different data sources. Median IAP was calculated as the median of each patient's first 48 h of postoperative IAP unless a different postoperative period is explicitly specified. When stratifying by subgroups, the median IAP per patient was further aggregated to the median per subgroup.

Furthermore, we used two approaches to explore the duration of IAH. *Cumulative* IAH was defined as the total time (in hours) that a patient spent at a given IAH grade or greater. We defined *consecutive* as the maximum consecutive time (in hours) that a patient spent at a given IAH grade or greater. Time was still considered consecutive if it was not interrupted by more than 30 min of a lower IAH grade (including no IAH). Patients may experience higher IAH grades but will also be reflected in lower IAH grades.

Our primary aims were descriptive, so we did not use a formal hypothesis test and, therefore, sample size calculation. Instead, we used a sample of ongoing registry study patients.

Data preparation and analysis were carried out using the python language with support from the following packages: crcmod 1.7, numpy 1.19.1 [31], boto3 1.16.0, matplotlib 3.3.1 [32], pandas 1.1.3[33], psycopg2 2.8.5, pylint 2.6.0, pytest 6.1., python-graphviz 0.14.2 [34], scipy 1.5.2 [35], sqlalchemy 1.3.19 [36], openpyxl 3.0.5, rpy2 3.3.6 and statsmodels 0.12.0.

## Results

**Fig. 2:**
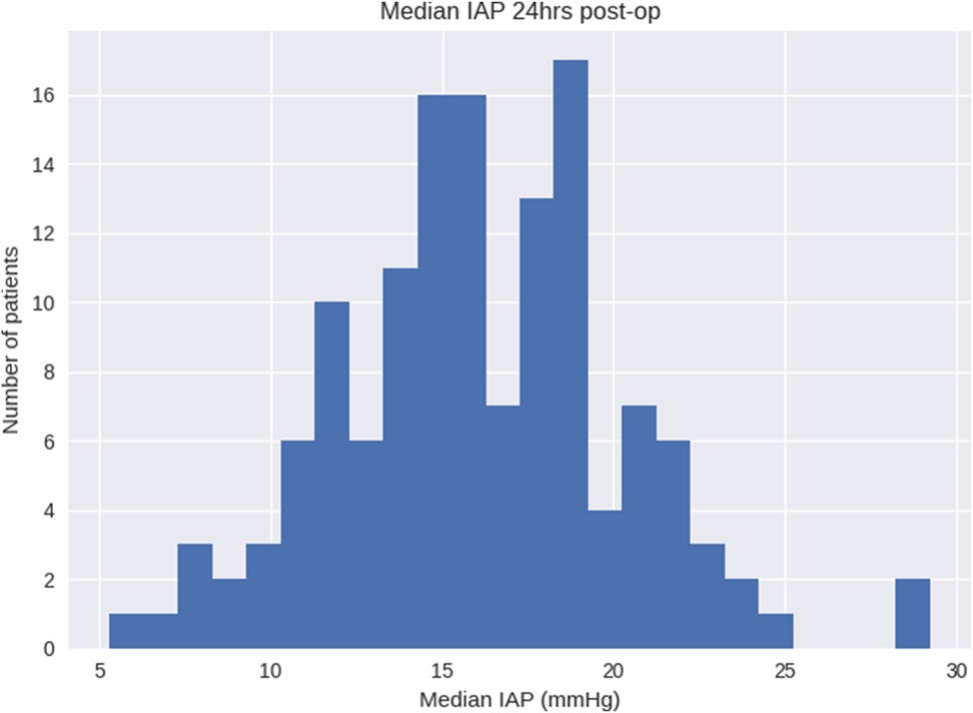
Histogram of median intra-abdominal pressure from surgery end to 24 h postoperatively. The median IAP within the first 24 postoperative hours was 15.9 [13.6, 18.7] mmHg. *IAP* intra-abdominal pressure; *mmHg* millimeter Hydrargyrum (Mercury)

**Fig. 3:**
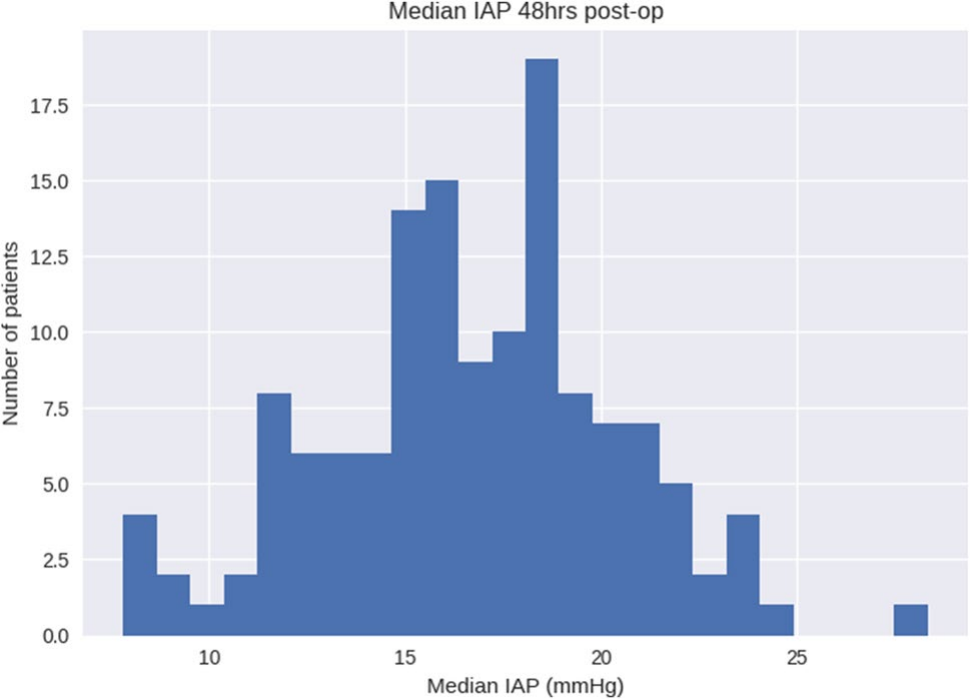
Histogram of median intra-abdominal pressure duration within the time period from 24 to 48 postoperative hours. The median IAP within this time frame was 16.6 [14.5, 19.1] mmHg. *IAP* intra-abdominal pressure; *mmHg* millimeter Hydrargyrum (Mercury)

**Fig. 4:**
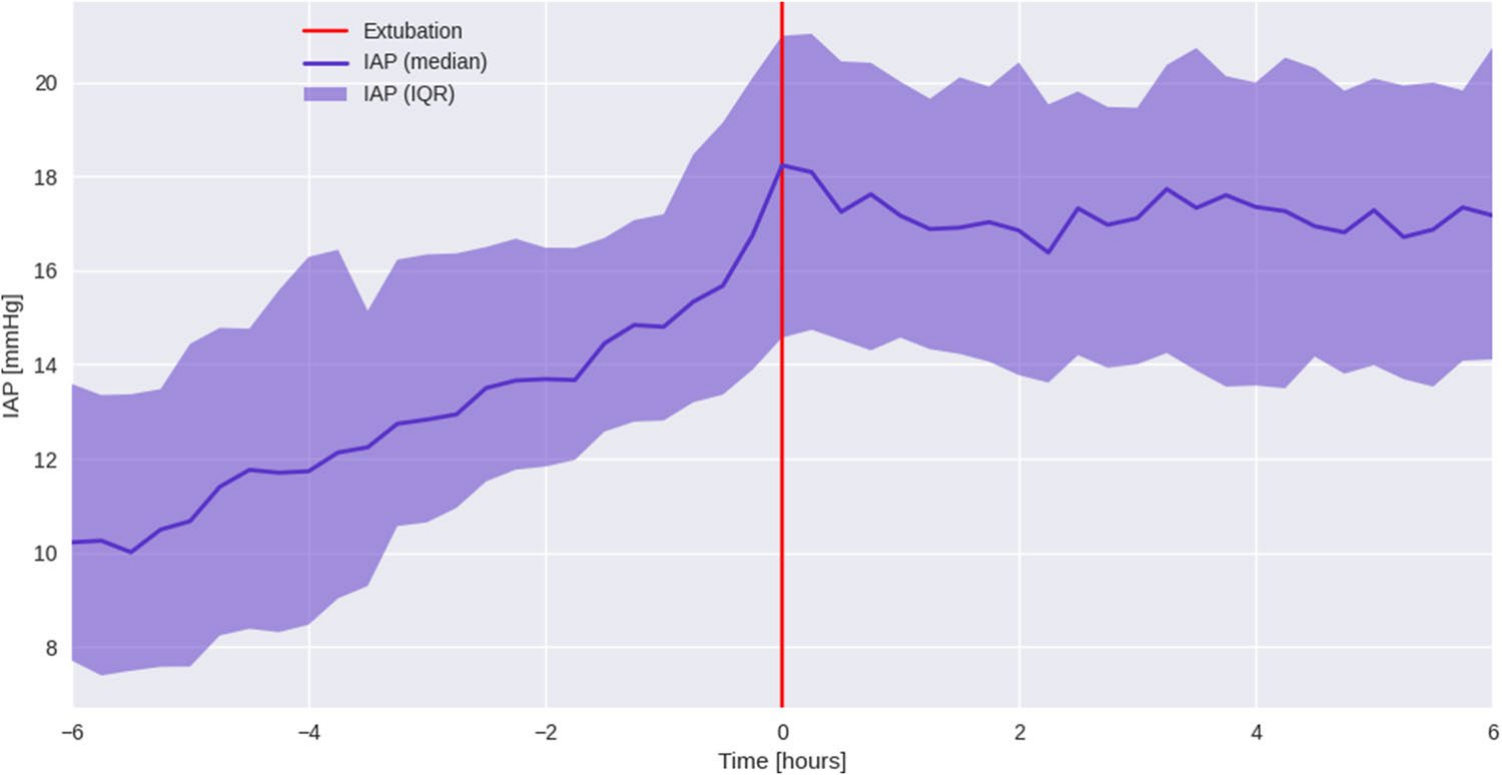
Course of median IAP 6 h before and after extubation. Red line represents time of extubation. Median IAP within the 6 h’ time frame before extubation is 10.2 [7.7, 13.6] and 17.2 [14.1, 20.7] after extubation

**Fig. 5:**
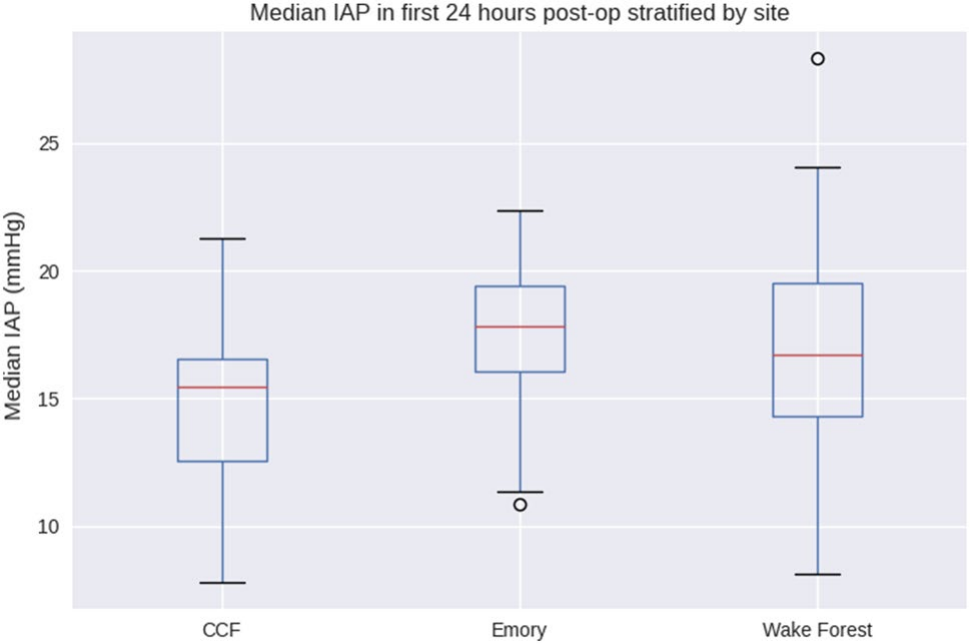
Differences in IAP by CVICU site. Median [IQR] for CCF 15.4 [12.5, 16.5], Emory 17.8 [16.0, 19.4], Wake Forest 16.7 [14.3, 19.5]. CCF, Cleveland Clinic Florida, Emory University Hospital Midtown and Wake Forest Hospital. CVICU, cardiovascular intensive care unit

**Fig. 6:**
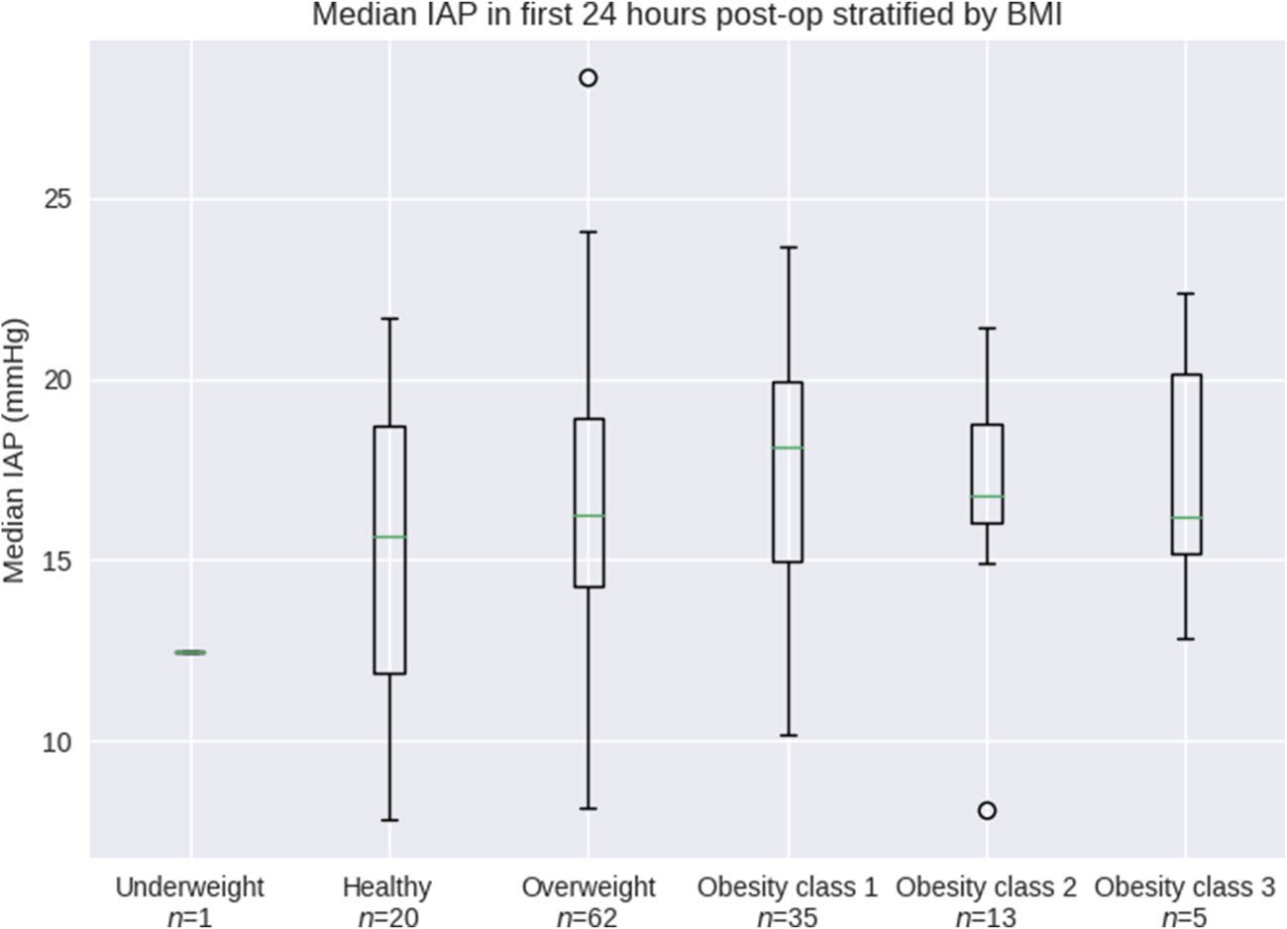
Differences in IAP by body mass index (BMI) within the first 24 postoperative hours. Median [IQR] for different BMIs are reflected as follows: Underweight: 12.5 [12.5, 12.5], healthy weight: 15.7 [11.9, 18.7], overweight: 16.3 [14.3, 18.9], obesity class 1: 18.1 [15.0, 19.9], obesity class 2: 16.8 [16.0, 18.8], obesity class 3: 16.2 [15.2, 20.1]. Underweight = BMI of < 18.5, healthy weight = BMI of 18.5 to < 25, overweight = BMI of 25.0 to < 30, obesity class 1 = BMI of 30 to < 35, obesity class 2 = BMI of 35 to < 40, obesity class 3 = BMI of 40 or higher. IAP, intra-abdominal pressure

This analysis included 173 cardiac surgery patients from three academic centers. We excluded patients with Foley catheter dwell times < 24 h (36 patients), with 137 patients qualifying for final analysis. Patient demographics, surgical characteristics, and levels of perioperative hemodynamic support are shown in Tables 1, 2, and 3. The Accuryn Monitoring System took a median of 20,640,100 [17,460,000; 27,931,600] bladder pressure measurements per patient. In total, 3,688,161,000 (> 36^8) bladder pressure measurements during 427 patient days were recorded. Baseline IAP was a median of 6.3 [4.0, 8.1] mmHg. 100% (137/137) of patients displayed IAH (of at least grade I) at one or more time points during the study period. The median IAP within the first 24 postoperative hours was 15.9 [13.6, 18.7] mmHg and the median IAP in the following 24–48 h was 16.6 [14.5, 19.1] mmHg (Figs. 2 and 3). Median extubation time (calculated from surgery end) was 325 min [239, 750]. The time course of IAP centered around extubation is shown in Fig. 4. The median IAP within a 6 h’ time frame before extubation was 10.2 [7.7, 13.6] and 17.2 [14.1, 20.7] after. Extubation data were only available for 115/137 patients. IAP stratification by demographic variables such as gender, procedure type and on/off CPB are shown in Table 4 whereas IAP stratification by obesity classes and study sites are depicted in Figs. 5 and 6.

**Table 1:** Characteristics of patients after cardiac surgery

Covariate	Overall (N = 137)
Age (years), median [IQR]	66.4 [58.3, 72.0]
BMI (kg/m2), median [IQR]	28.9 [26.0, 32.0]
Female/ Male (%)	54 (39.4)/83 (60.6)
Race (%)	
White or Caucasian	109 (79.6)
Black or African American	23 (16.8)
NA	3 (2.2)
Other	2 (1.5)
Ethnicity	
Non-Hispanic or Latinx	130 (94.9)
Hispanic or Latinx	4 (2.9)
Site	
Wake Forest	88 (64.2)
Emory	29 (21.2)
CCF	20 (14.6)
Baseline IAP [mmHg], median [IQR]	6.3 (4.0, 8.1)
0–24 postop hours: IAP [mmHg], median [IQR]	15.9 [13.6, 18.7]
24–48 postop hours: IAP [mmHg], median [IQR]	16.6 [14.5, 19.1]

Values are represented as median and interquartile ranges [IQR] or in %

*BMI* body mass index; *kg/m*^*2*^ kilogram/meter squared; *CCF* Cleveland Clinic Florida; *IAP* intra-abdominal pressure; *mmHg* millimeter Hydrargyrum (Mercury); *postop* postoperative

**Table 2:** Surgical characteristics

Covariate	N (%)/median [IQR]
Type of surgery	
CABG surgery	54 (39.4)
Valve surgery	40 (29.2)
CABG plus valve surgery	10 (7.3)
OFF pump CABG surgery	9 (6.6)
Other cardiac surgery	7 (5.1)
Cardiac Surgery including support devices	6 (4.4)
Redo cardiac surgery	5 (3.6)
CABG plus non-valve surgery	4 (2.9)
Heart Transplants	2 (1.5)
Procedures with CPB	126 (92)
CPB duration [h], median (IQR)	1.9 [1.3–2.4]
Surgery duration [h], median (IQR)	5.0 [4.5–6.2]
Foley dwell duration [h], median (IQR)	56.0 [46.8–77.5]
Duration of IAP above 20 mmHg [h], median [IQR]	3.8 [0.2–8.8]

Values are represented as median and interquartile ranges [IQR] or in %

*CABG* coronary artery bypass graft; *CPB* cardiopulmonary bypass; *IAP* intra-abdominal pressure

**Table 3:** Hemodynamic support types and blood transfusion characteristics

POD	Support type	Support level, % (N)		
		Low (0)	Medium (1–2)	High ($\ge$ 3)
0	Vasoactive drugs			
	Inotropes	35.0% (48)	62.8% (86)	2.2% (3)
	Vasopressors	0.7% (1)	83.9% (115)	15.3% (21)
	Blood products			
	Cryoprecipitate (bags)	92.7% (127)	5.1% (7)	2.2% (3)
	FFP (bags)	91.2% (125)	4.4% (6)	4.4% (6)
	Platelets (bags)	73.0% (100)	14.6% (20)	12.4% (17)
	pRBC (bags)	78.1% (107)	8.0% (11)	13.9% (19)
1	Vasoactive drugs			
	Inotropes	55.5% (76)	44.5% (61)	0
	Vasopressors	41.6% (57)	56.2% (77)	2.2% (3)
	Blood products			
	Cryoprecipitate (bags)	97.8% (134)	1.5% (2)	0.7% (1)
	FFP (bags)	97.8% (134)	1.5% (2)	0.7% (1)
	Platelets (bags)	94.9% (130)	2.2% (3)	2.9% (4)
	pRBC (bags)	84.7% (116)	6.6% (9)	8.8% (12)

Values are represented as in % or absolute numbers in parenthesis. Low (0), Medium (1–2) or High ($\ge$ 3) support of vasoactive drugs represent the number of inotropes of vasopressors that patients were receiving. Inotropes represent Dobutamine, Milrinone, Epinephrine, Dopamine. Vasopressors represent Norepinephrine, Vasopressin, Phenylephrine, Angiotensin II

*POD *postoperative day; *CABG* cardiopulmonary bypass graft; *FFP* fresh frozen plasma; *pRBC* packed red blood cells

**Table 4:** Median IAP [IQR] is described for different patient and surgical covariates

Covariate	IAP (mm Hg), median [IQR]
Gender	
Female	17.9 [14.2, 19.6]
Male	16.4 [14.7, 18.9]
Type of surgery	
CABG surgery	16.8 [14.9, 19.6]
Valve surgery	16.3 [13.6, 18.6]
CABG plus valve surgery	17.4 [15.3, 18.8
OFF pump CABG surgery	16.1 [15.7, 20.6]
Other cardiac surgery	18.9 [15.9, 19.5]
Cardiac Surgery including support devices	14.8 [14.3, 15.1]
Redo cardiac surgery	18.8 [18.4, 19.2]
CABG plus non-valve surgery	19.3 [16.9, 20.3]
Heart transplants	11.7 [9.9, 13.5]
Surgical procedure ON/OFF pump	
ON -pump (CPB)	16.6 [14.4, 18.9])
OFF -pump	16.1 [15.4, 19.9])

Values are represented as median and interquartile ranges [IQR]

*IAP* intra-abdominal pressure; *mmHg* millimeter Hydrargyrum (Mercury); *CABG* cardiopulmonary bypass; *CPB* cardiopulmonary bypass

### Time course of IAH

IAP was measured beginning with induction of anesthesia and continued intraoperatively and after that for a median of 56.0 [46.8, 77.5] hours postoperatively. Intraoperatively (1 h or more before the end of surgery), most patients, 77% (105/137) remained within a normal IAP (< 12 mmHg) range. 56% (77/137) of patients had IAH by 1 h after surgery with a median IAP of 12.5 mmHg [10.0, 15.3] mmHg, increasing 86% (118/137) of patients at 6 h after surgery with a median IAP of 15.0 [12.4, 18.9], and the median IAP remained elevated throughout the first 48 h with a median of 17.8 [14.9, 20.4] (Fig. 1). Urine output peaked just before the end of surgery and decreased quickly afterward with median values of 1.2 [0.7, 2.5] ml/kg/h at 1 h, 0.6 [0.4, 0.9] ml/kg/h at 6 h and 0.5 [0.3, 0.6] ml/kg/h at 12 h after surgery end.

### Cumulative duration of IAH

The maximum cumulative duration of IAH (grades I–IV) broken up into various postoperative periods (0 -12, 0–24, 0–36 and 0–48 postoperative hours) is shown in Supplemental Fig. 1a–d. For example, out of 48 postoperative hours (Supplemental Fig. 2d), 100% (137/137) of patients spent at least 1 h, 99% (136/137) spent at least six and 93% (128/137) more than 12 h in IAH grade I. 98% (134/137) of patients spent at least 1 h, 90% (123/137) at least six and 82% (113/137) at least 12 h of cumulative time in IAH grade II. IAH grade III was recorded in 78% (107/137) of patients for more than one, 55% (75/137) more than six and 39% (53/137) more than 12 h. And lastly, 36% of patients (50/137) spent more than 1 h in IAH grade IV, 12% (17/137) more than 6 h and 5% (7/137) more than 12 h.

Detailing the progression of IAH during the first 48 postoperative hours, 93% (128/137) of patients spent at least 12 h in IAH grade I, 88% (113/128) of those patients in grade I also had grade II, 47% (53/113) of patients with grade II also had grade III, and 13% (7/53) of patients with grade III also had grade IV IAH.

### Maximum consecutive duration of IAH

The maximum consecutive duration of IAH (grades I–IV) parted into various postoperative periods (0–12, 0–24, 0–36 and 0–48 postoperative hours) is shown in Supplemental Fig. 1a–d. For example, out of 48 postoperative hours (Supplemental Fig. 1d), 100% (137/137) of patients spent at least 1 h, 93% (128/137) at least six, and 84% (115/137) at least 12 consecutive hours in IAH grade I. IAH grade II was experienced by 96% (132/137) of patients for more than one, 81% (111/137) for more than six, and 62% (85/137) for more than 12 h. 75% (103/137) spent at least 1 h, 38% (52/137) at least 6 h, and 18% (25/137) at least 12 h in IAH grade III. Lastly, 37% (51/137) of patients spent more than 1 h, 4% (5/137) more than six, and 2% (3/137) more than 12 h in IAH grade IV (Supplemental Fig. 1d).

During the first 48 postoperative hours, 84% (115/137) of patients spent at least 12 consecutive hours in IAH grade I, 74% (85/115) of patients with grade I had grade II, 29% (25/85) of patients with grade II also had grade III, and 12.0% (3/25) of patients with grade III also had grade IV.

### IAP in patients with less than 24 h of IAP observations

34/36 patients had IAP observations for more than 12 h. Two patients had either left the ICU or the Foley catheter pulled before reaching a 12-h observation period. 79% (27/34) of patients spend more than 12 consecutive hours in IAH grade I, 56% (15/34) in grade II, and 27% (4/34) in grade III. Whereas 85% (29/34) of patients spent more than 12 cumulative hours in IAH grade I, 86% (25/34) in grade II, 20% (5/34) in grade III.

## Discussion

The present study is the first multicenter prospective observation on the incidence of IAH in cardiac surgery patients admitted to the ICU using a novel high-frequency measurement system. The results detail the trajectory of IAP both intra and postoperatively, highlighting that IAH is common and persistent in this patient population. The incidence of any grade of IAH (for at least 6 h per patient) during the whole study period (48 h) was 99.3%. Therefore, when IAP is measured continuously, IAH is detected much more commonly than the traditional intermittent spot check measurements reported in only a few small single-center studies reporting IAH at 33–46% [22–24, 37].

Measuring IAP has traditionally been performed by instillation of normal saline into the bladder through a Foley catheter and then taking hydrostatic pressure measurements of bladder pressure. These methods use either a fluid column or a transducer setup to obtain the bladder pressure with the patient in the supine position. These systems are zeroed at the patient’s midaxillary line at the level of the iliac crest [7]. During surgery, these manual IAP recordings are not feasible. Even postoperatively in the ICU setting, this method is laborious and time-consuming and only performed when there is a high clinical suspicion for intra-abdominal hypertension or an abdominal compartment syndrome. Furthermore, these IAP measurements are imprecise and highly operator-dependent, specifically in the setting of IAH [38].

Both the frequency of IAP monitoring and the definition of IAH (times of elevated IAP measurements) varies across published literature. Studies involving cardiac surgery patients measured IAP at the shortest time interval of 3 h (once) and the longest interval of 12 h [22–24, 37]. The frequency of IAP measurement recommended by the WSACS is a baseline and every 4–6 h (or continuous) if two or more risk factors for IAH are present. Furthermore, WSACS defines IAH by a sustained or repeated pathological elevation in IAP ≥ 12 mmHg. In the consensus guidelines, maximal IAP measurement was suggested, although consideration is given to using mean or median values of consecutive measurements [7]. However, this definition leaves room for interpretation which is further reflected in various studies evaluating IAH. For example, Kiliz et al., in a small single-center study of 100 subjects, counted patients as having IAH when all IAP measurements after the baseline were ≥ 12 mmHg. They measured IAP after anesthesia induction and then upon ICU arrival and at 12 and 24 h postoperatively [37]. Other authors defined the instance of IAH as single elevated IAP measurements and sustained IAH with two or more consecutive IAP measurements ≥ 12 mmHg (not defining the time interval) [3, 4, 22–24]. The largest multicenter study (491 patients from 15 medical-surgical ICUs) calculated the mean IAP for each patient on each study day and diagnosed patients with IAH if they had at least 1 day with a mean IAP of 12 mmHg or higher. This study further defined the maximum IAH grade based on the highest mean IAP of any study day [1].

Importantly the dynamics of IAH in cardiac surgery patients have not been well described due to the lack of frequent, automated IAP monitoring. Here our work is uniquely poised to be the first near-continuous estimation of intra-abdominal pressure in prospectively enrolled patients. While our sample size (137 patients) is not very different from other reported literature, we have pressure measurements more than any other available work, by many thousands of orders of magnitude (> 36^10 bladder pressure measurements during 427 patient days).

In our study, most patients displayed “normal” IAP (< 12 mmHg) during surgery, followed by a steep increase within the first five postoperative hours, and maintained high IAP measurements for 48 h (Fig. 1). Opposed to our findings, a recent single-center observational study of 191 cardiac surgery patients found IAH to be more prevalent during cardiac surgery than postoperatively with 105/191 (55%) displaying IAH after induction of anesthesia, 115/191 (60%) after chest closure, and 31/131 (24%) 2 h after arrival in the ICU. IAP was measured manually at these three-time points with paralysis ensured during the surgery [29]. In this study, IAP interestingly seems to increase until extubation and plateauing thereafter (Fig. 4). As to why this might be the case we can only speculate. For example, patients are only extubated when clinically stable and likely ready for or in the process of de-resuscitation or positive pressure ventilation that can contribute to increased IAP is ceased.

The incidence of IAH in our study is higher than previously described in the cardiac surgery population, with 100% of patients exhibiting at least one measurement of IAH grade I compared to 33–49% in previous studies [22–24, 37]. Only one study evaluated IAH grading, reporting 12% of patients in grade II IAH post-cardiac surgery (13/108 patients), grade II being the highest grade of IAH observed [23].

We qualitatively looked at the UO in relation to IAP (Fig. 1). In this postoperative timeline, UO appears lower with increased IAP during the study period, with higher UO seen intraoperatively than during the postoperative time in the ICU. Low UO is a hallmark of acute kidney injury (AKI), and KDIGO defines AKI as UO less than 0.5 ml/kg/h for more than 6 h (stage I) [39]. AKI is a frequent form of IAH- induced organ dysfunction [12, 40]. In patients with ACS, AKI is established with anuria and the necessity for renal replacement therapy unless early intervention prevents these sequelae [41]. A recent meta-analysis established the link between AKI and IAH in various patient populations including cardiac surgery [42].

The strengths of our study include the very large number of samplings of IAP within a pragmatically enrolled and other asymptomatic patient cohort studied across three academic sites and allowing for generalizability in therapeutic management principles in the post-cardiac surgery patient. This is the first study that uses the novel Accuryn Monitoring System automated IAP and CUO measurement system. The trending of frequent, repeated IAP measurement allows for greater confidence in IAP (lack of user dependence) and improved visibility of IAP trends. The precision of the Accuryn Monitoring System facilitates IAP measurements to the decimal point and challenges the grading of IAH by WSACS. For example, IAH grade I is defined as IAP 12–15 mmHg while grade II IAH is 16–20 mmHg leaving 15.1–15.9 mmHg essentially uncategorized. While UOP has not been a focus of this study, automated CUO measurements have the same advantages in early detection of low UO enabling earlier interventions. Limitations of this descriptive qualitative study include its observational design and small sample size, which may preclude any direct inferences to causality and hence interventional options. While the patient sample size is still relatively small, the system captured a median of over 20^10 individual measurements per patient. Specifically, the current study represents the largest available descriptive snapshot of this patient cohort in cardiac surgery. Secondly, patient positioning, sedation, paralysis, and optimal conditions for IAP measurements are not accounted for. Having said that, we did not see any abrupt fluctuations in measurements that would be typical of the straining patient with artifact-related high IAP. While measurements of IAP in the ICU are increasingly taken in the “head of bed up by 30 degrees” position (HOB 30), the formal definition of IAH still requires patients to be in the supine position [7]. However, we also adjusted the IAP measurements for artifacts due to positional changes or coughing by utilizing a running 10-min IAP median. Third, while the Accuryn Monitoring System was bench validated in comparison to calibrated laboratory pressure sensors (Supplement), no comparison to the currently conceived “gold standard” of intermittent bladder pressure measurements through a hydrostatic column or transducer has been performed. Fourth, the timeframe for the study is limited to 48 h, although the median Foley catheter dwell time was 56.0 h [46.8, 77.5], and IAP appeared elevated for 45.2 [29.2, 65.8] hours (note that if the catheter is removed while IAP is elevated the data will be right-censored). The 48 h period was chosen because 79% (137/173) of patients remained in the study and were available for analysis during this time frame. Fifth, as the database is still incomplete (ongoing prospective observational study) data remain for this analysis limited. We do not have the granularity to report medical history, fluids or infusion rates of vasoactive medications. In lieu of these data, we chose to report different support types acknowledging that we do not adjust for infusion rates to give an imperfect perspective of the analyzed patient population. Sixth, this observational study is relying on the documentation into the EMR which rarely is done real time (if at times at all) which can lead to missing data points and misalignment of real-time data with documentation. And finally, we do not yet have any associations to adverse outcomes such as acute kidney injury. In future studies, we plan to evaluate a possible correlation between IAH and AKI in detail. Our present report may therefore be conceived as pilot data from an ongoing registry that continues to enroll. It may be important to detect low-grade IAH early to prevent the development of higher grades of IAH and ACS. IAH is associated with pathophysiological changes and increased morbidity and mortality [1, 23, 43]. Future analysis of continuous abdominal perfusion pressure in the setting of continuous IAP and systemic blood pressure measurements is necessary to provide further evidence for mechanisms of clinical outcomes and organ system injury.

In conclusion, our data highlight that IAH is common and persistent in cardiac surgery patients. Improved and automated monitoring of IAP will increase the detection of IAH—which normally would remain undetected using traditional monitoring methods.

## Supplementary Information

Below is the link to the electronic supplementary material.

Supplementary file1 (DOCX 13770 kb)

## Abbreviations

IAH: Intra-abdominal hypertension
CUO: Continuous urinary output
UO: Urinary output
IAP: Intra-abdominal pressure
ACS: Abdominal compartment syndrome
WSACS: The Abdominal Compartment Society
ICU: Intensive care unit
BMI: Body mass index
AKI: Acute kidney injury
KDIGO: Kidney Disease: Improving Global Outcomes
