# Supplementary material for: Intra-abdominal hypertension in cardiac surgery patients: a multicenter observational sub-study of the Accuryn registry
Source: J Clin Monit Comput. 2022 Jun 13;37(1):189–99. doi: 10.1007/s10877-022-00878-2 (PMC9852117; doi:10.1007/s10877-022-00878-2)

## **Supplemental Figures:**

**Supplemental Figures 1 a – d:** The *cumulative* duration above different IAP thresholds is displayed within different time frames: a) Cumulative duration of IAH within 12 postoperative hours, b) Cumulative duration of IAH within 24 postoperative hours, c) Cumulative duration of IAH within 36 postoperative hours, d) Cumulative duration of IAH within 48 postoperative hours. Intra-abdominal pressure; IAH, intra-abdominal hypertension; mm Hg, millimeter Hydrargyrum (Mercury).

a) Cumulative duration of IAH within 12 postoperative hours


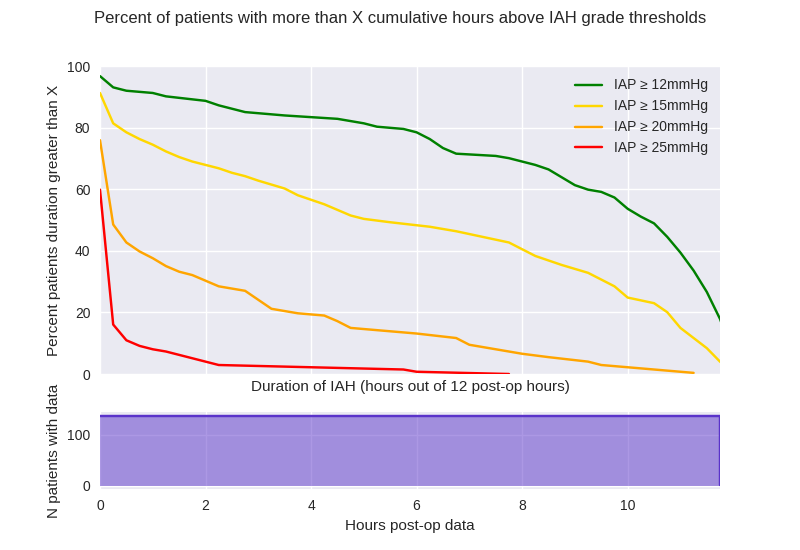


b) Cumulative duration of IAH within 24 postoperative hours


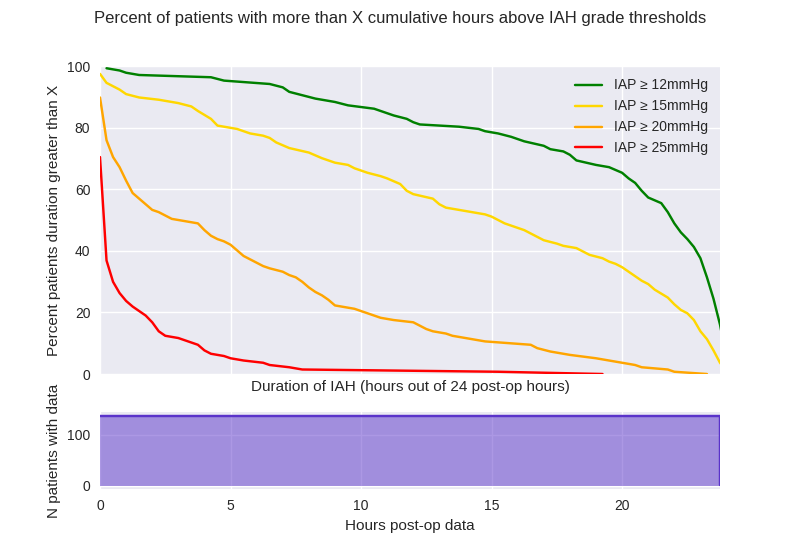


c) Cumulative duration of IAH within 36 postoperative hours


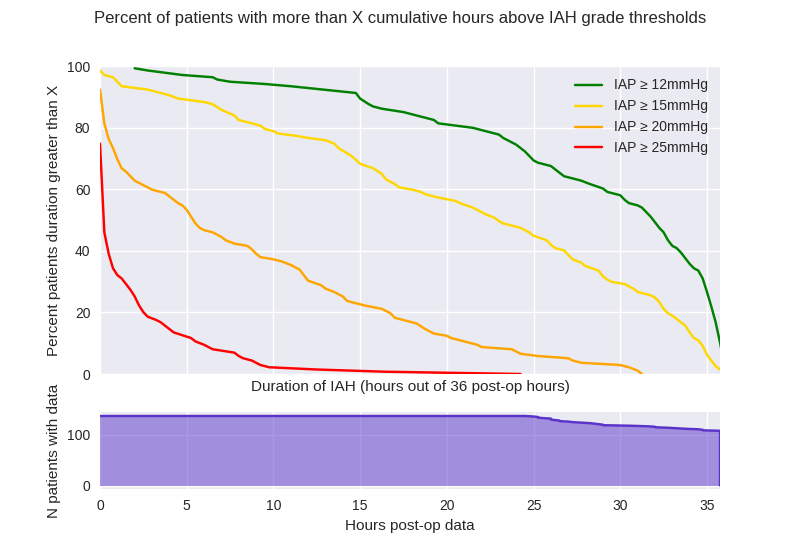


c) Cumulative duration of IAH within 48 postoperative hours


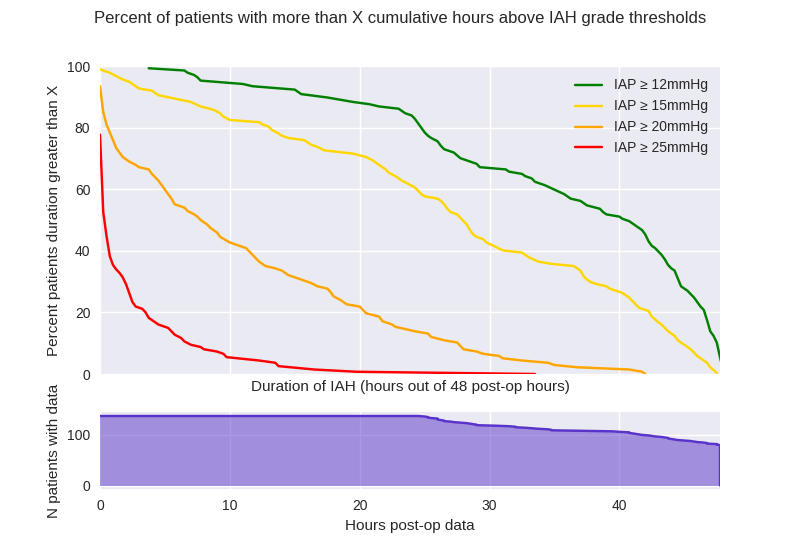


**Supplemental Figures 2 a – d:** The *consecutive* duration above different IAP thresholds is displayed within different time frames: a) Consecutive duration of IAH within 12 postoperative hours, b) Consecutive duration of IAH within 24 postoperative hours, c) Consecutive duration of IAH within 36 postoperative hours, d) Consecutive duration of IAH within 48 postoperative hours. Intra-abdominal pressure; IAH, intra-abdominal hypertension; mm Hg, millimeter Hydrargyrum (Mercury).

a) Consecutive duration of IAH within 12 postoperative hours


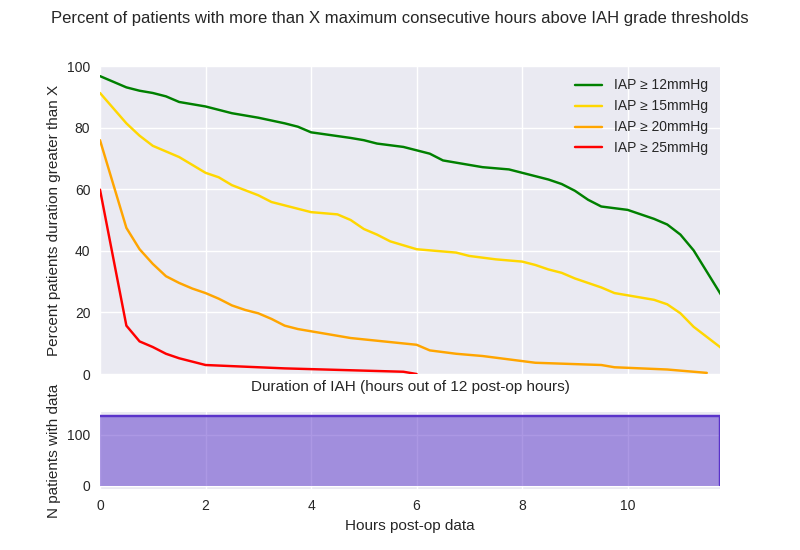


b) Consecutive duration of IAH within 24 postoperative hours


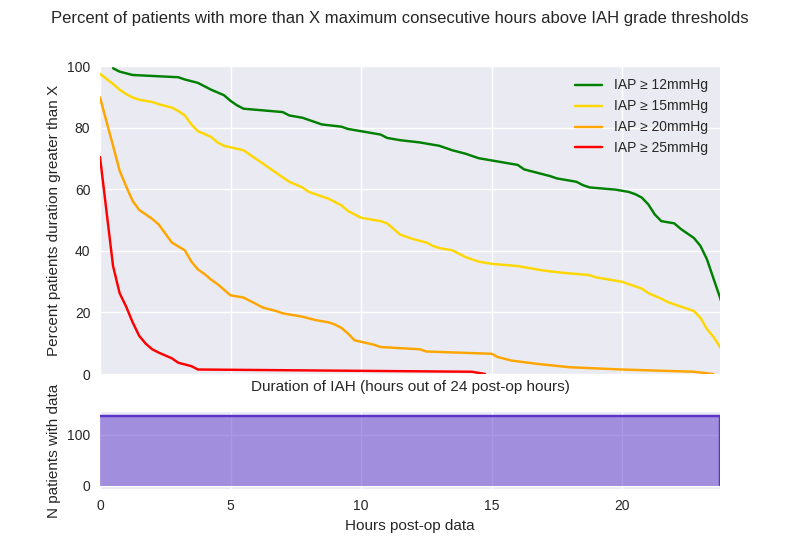


c) Consecutive duration of IAH within 36 postoperative hours


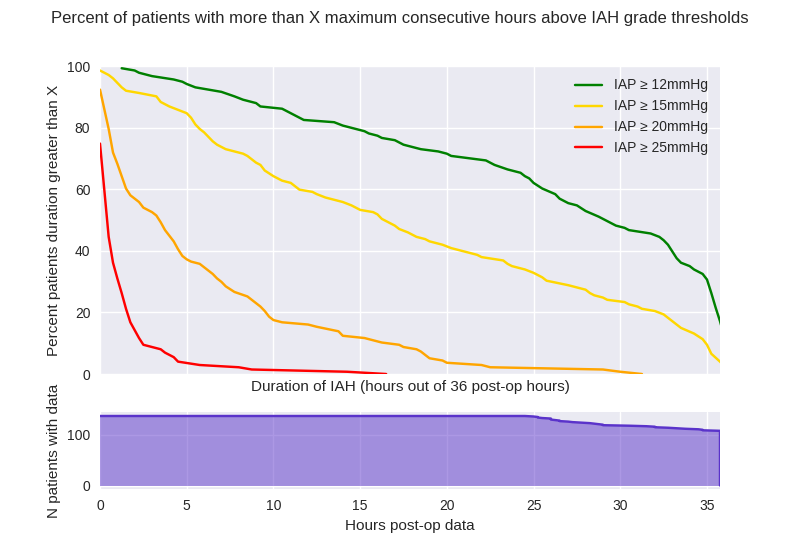


d) Consecutive duration of IAH within 48 postoperative hours


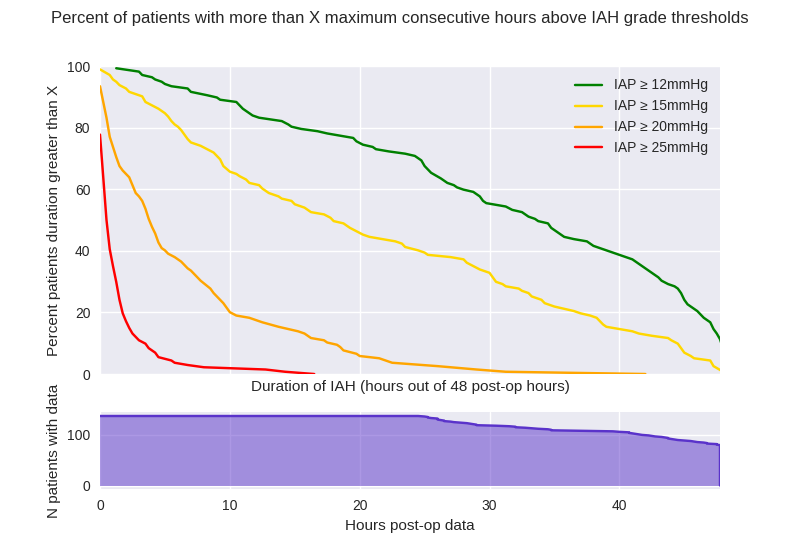

Supplement: Supplementary file 1 — Supplementary file1 (DOCX 13770 kb) [file 10877_2022_878_MOESM1_ESM.docx]
